# Molecular Mechanisms of Bioactive Nutrients Promoting Health through Gut Microbiota

**DOI:** 10.3390/ijms241813731

**Published:** 2023-09-06

**Authors:** Baojun Xu

**Affiliations:** Food Science and Technology Program, Department of Life Sciences, BNU-HKBU United International College, Zhuhai 519087, China; baojunxu@uic.edu.cn

Many food components (such as phytochemicals, complex carbohydrates, proteins, fats, vitamins, minerals, etc.) have been found to have various biological activities. Based on dietary intake and the availability of nutrients in the intestine, human gut microbiota can produce harmful metabolites that cause human diseases or beneficial compounds that prevent host diseases [1]. Abnormal gut microbiota can produce endotoxins, exacerbating chronic inflammation and metabolic disorders [2]. Moreover, gut microbiota are crucial for maintaining metabolism and health, and dysbiosis plays a crucial role in the occurrence and development of various diseases [3].

Therefore, a promising strategy to help manage colon and host health is to regulate the composition of the gut microbiota by eating biologically active food ingredients. Bioactive ingredients obtained from dietary sources can be designed and characterized to meet human nutritional and immune needs and balance gut microbiota. To maximize knowledge on the health effect of gut microbiota on improving human health, a Special Issue titled “Molecular Mechanisms of Bioactive Nutrients Promoting Health through Gut Microbiota” was published in the *International Journal of Molecular Science*, including nine papers: six research articles and three reviews. Among these six research articles, four are animal studies [4,5,6,7], one is in vitro gut microbiota culture study [8], and one is randomized clinical study [9].

Vernocchi et al. [1] summarized the metabolism of gut microbiota and their interactions with food components, including the dietary impact on gut microbiota and metabolic composition, health effects mediated by food–microbiota metabolomes, and microbiome-based therapeutics. Finally, the author also pointed out that future research requires extensive experiments, and the role of specific nutrients needs to be evaluated in clinical trials.

The review by Mercader-Barceló et al. [10] recorded that regulating dietary factors can affect the development of idiopathic pulmonary fibrosis (IPF) through the intestinal–pulmonary axis. This review summarized evidence about the relationship between diet and IPF in human and pulmonary fibrosis animal models. The authors also discussed the biological activities of specific dietary food ingredients, including fatty acids, peptides, amino acids, carbohydrates, vitamins, minerals, and phytochemicals. Moreover, future research should aim to identify novel diet-related biomarkers of IPF, including metabolites derived from microbiota, and conduct a more in-depth analysis of lung and intestinal microbiota in IPF.

Ganesan et al. [11] summarized the treatment of colorectal cancer using diet-derived phytochemicals through colon cancer stem cells and microbiota. It also reviewed the regulation of different phytochemicals on gut microbiota through different molecular mechanisms and proposed a relationship between phytochemicals, gut microbiome, and colon cancer stem cells. The authors suggested that bioactive nutrients will improve the gut microbiota and combat colorectal cancer. In the end, the authors suggested that dietary phytochemicals-induced intestinal microbiota are still a potential research field because they have an apparent anti-tumor effect and are a new mechanism for future treatment.

An animal study [6] has shown that pistachios can reduce inflammation and improve gut microbiota composition in mice consuming a high-fat diet. Specifically, pistachios can significantly reduce the protein levels of TNF-α and IL-1β in sera and the mRNA expression levels of IL-1β, TNF-α, F4-80, and CCL-2 in subcutaneous and visceral adipose tissues. A treatment with pistachios can reduce the ratio of *Firmicutes* to *Bacteroidetes*. Furthermore, the pistachio diet can significantly increase the abundance of healthy bacteria genera, such as *Parabacteroides, Dorea, Allobaculum, Turicibacter, Lactobacillus,* and *Anaeroplasma*. It considerably reduced the amount of inflammation-related bacteria, such as *Oscillospira, Desulfovibrio, Coprobacillus,* and *Bilophila*. Terzo et al. [6] proposed that the consumption of pistachios can alleviate inflammation caused by obesity, and this effect may be related to the regulation of microbial community composition.

Complex carbohydrates have been widely used in the food industry, but their positive effect ton gut microbiota and the related mechanisms are still unclear. Wang et al.’s [5] animal experimental studies have shown that chitosan can improve the colitis of dextran sulfate sodium-induced ulcerative disease by enhancing the internal barrier function and improving microflora. Chitosan can also improve the intestinal mucosal barrier function and affect gut microbiota. It can also better regulate the expressions of TNF-α and TJ proteins, such as claudin-1, occludin, and ZO-1. Moreover, Liang et al.’s [4] study revealed that d-Tagatose can improve constipation by modulating the composition of gut microbiota. More specifically, taking d-Tagatose can restore changes in gut microbiota caused by constipation, significantly increase level of acetylcholine (Ach) in sera, and also reduce the level of nitric oxide (NO) in sera. Nogacka et al. [8] conducted fecal culture tests in vitro on the microbiota of healthy adults with a normal weight and morbidly obese adults, with the addition of different inulintype fructans (1-kestose, actilight, P95, synergy1, and inulin) and a galactooligosaccharide to the cultures. The regulation of prebiotics leads to a significant increase in the numbers of *Bacteroides, Bifidobacterium,* and *Faecalibacterium.* The author suggests that there are differences in the tested prebiotics among different populations, indicating that research and development need to be tailored to specific populations when developing related products.

Diseases are often closely related to changes in gut microbiota composition, and unraveling the relationship may identify new molecular mechanisms for the subsequent treatment of diseases. Nagpal et al. [7] explored the relationship between gut microbiota and obesity through three commonly used mouse models of obesity or type 2 diabetes. The results indicate that obesity caused by a high-fat diet and gene mutations exhibit different gut microbiome compositions, indicating that the microbiome is sensitive to the hosts’ diet, genetic background, and physiology.

When studying the relationship between diseases and gut microbiota, in addition to using animal models, it is also necessary to study human gut microbiota. Tomova et al. [9] explored the impact of food intake specificity on gut microbiota among children with autism. The dietary intake of children with autism provides the same energy as the normal, but their food choices may lead to a lack of micronutrients, such as vitamin K, B6, C, iron, copper, docosahexaenoic acid, and docosapentaenoic acid. Food selectivity and the intake of fermented milk products, total fat, omega-3, and animal/plant protein lead to similar changes in the intestinal microbiota of children with and without autism. The authors pointed out that although food intervention is difficult for children with autism, such changes may help alter gut microbiota, thereby improving their gastrointestinal and immune states.

As researchers continue to conduct studies on the role of gut microbiota and their metabolites in diseases, research has gradually shifted from non-targeted therapy to targeted microbiota therapy [2] using new technical strategies like fecal microbiota transmission (FMT), thereby treating diseases more efficiently [11]. Moreover, the latest research has been published. Wang et al. [12] utilized an FMT experiment to target the potentially critical microbiota of hyperlipidemia. The causal relationship between changes in gut microbiota and lipid metabolism has been determined, and the mechanisms of Eucommia bark extract and Eucommia leaves extract in combating hyperlipidemia have been elucidated, and feasible treatment methods for hyperlipidemia have been provided. Yang et al. [13] explored the alleviating effect of Fu brick tea thearownin on ulcerative colitis and its potential mechanisms through fecal 16S rRNA genes, metabolomics, and FMT. Chen et al. [14] used FMT, 16S rRNA sequencing, miRNA sequencing, and RNA sequencing to elucidate the role of gut microbiota/butyric acid/miR-204/ACSS2 in regulating chicken fat production and deposition.

This Special Issue has not published any papers on transformation or targeted microbiota work, but it includes one clinical randomized trial. Subsequent research on modifying gut microbiota through bioactive nutrients to achieve health promotion needs to be carried out in more research on targeted microbiota therapies and clinical randomized trials. The relevant results of these targeted microbiota therapies and clinical randomized trials can be used to develop healthy products or drugs in the food or pharmaceutical industry.
